# Analysis on the geographical pattern and driving force of traditional villages based on GIS and Geodetector: a case study of Guizhou, China

**DOI:** 10.1038/s41598-023-47921-z

**Published:** 2023-11-24

**Authors:** Kehua Wu, Weici Su, Shi’an Ye, Wei Li, Yang Cao, Zhenzhen Jia

**Affiliations:** 1 Institute of Mountain Resources, Guizhou Academy of Sciences, Guiyang, 550001 China; 2 Guizhou Karst Cave (Tourism) Resources Development and Utilization Engineering Technology Research Center, Guiyang, 550001 China; 3 School of Geography and Tourism, Chongqing Normal University, Chongqing, 401331 China

**Keywords:** Ecology, Environmental sciences, Environmental social sciences

## Abstract

Traditional villages have received widespread attention from all walks of life based on important carriers of Chinese rural culture. The mutual superposition of natural and cultural factors may exacerbate the evolution of traditional village geographical patterns. To understand such relationships and effects, factors and degrees influencing traditional villages need to be determined. Here, we analysed the data of 724 traditional villages in Guizhou recognised by relevant national ministries and commissions in China using average nearest neighbour analysis, Tyson polygon analysis, nuclear density analysis and Geodector. The geographic pattern feature revealed that traditional villages, in general, are highly clustered regionally and have significant edge effects on administrative units. Different substrate environments result in significant spatial heterogeneity in village spatial density, clustering, surface undulation, sun exposure, and waterfront. The geographic pattern of traditional villages is mostly affected by the closest distance to river valleys, the types and number of intangible cultural heritage resources in the county, river gorge density, edge effect index, degree of county ethnic language use, and proportion of paddy fields to the regional area; and their combined effects influence and control the community structure. The results highlight the impact of nature and culture on the distribution of traditional villages, which helps traditional village conservation and scientific exploration of human-land relationship issues in the mountainous areas of Southwest China.

## Introduction

Traditional villages are heritage revitalisation carriers in both tangible and intangible forms, rich in historical, cultural, artistic, scientific, social, and economic values^1–6^, and are the basic units of blood and geo-relations knitted into relative independence^7^, engraved with the excellent genes of thousands of years in the progress of traditional farming civilisation, and played an very important role in the traditional rural culture protection. However, along with the rapid progress of modernisation and urbanisation, traditional villages are facing many problems, such as hollowing out^8^, landscape fragmentation^9^, lack of development sustainability^10^, disappearance of traditional customs, and loss of cultural functions^11,12^.

Rural settlements have always been the core and focus of rural geography research^13^, and as one of the special forms, traditional villages have received extensive attention, and their morphology, structure, function, and geographical differentiation can better indicate the footprint of human-nature interactions^14^.Scholars have conducted national-scale research on the geographic pattern of traditional villages through GIS, geostatistics, and other technical methods: the selected traditional villages of China in the early stage were mostly concentrated in areas with high economic levels, good transportation conditions, and a small proportion of minority population^15^,and there was a spatial difference of "south hot-north cold" that gradually decreased from southwest to northeast^16^ and later evolved into an "east‒west coexistence" pattern^17^, mostly concentrated at high altitudes, undulating terrain, fragmented surfaces, near the middle and upper reaches of rivers, far from urban centres and major grain-producing areas, with low transportation access and less economically developed agroforestry areas^18,19^. The Yangtze River, Pearl River, southeastern rivers and Yellow River are considered the major areas of traditional village distribution^20^. Studies on the spatial and temporal patterns and evolution of traditional villages in provinces and regions have also become more diverse, with hotspot areas focusing on Anhui^21^, Hunan^22,23^, Yunnan^24^, Zhejiang^25^, Jiangxi^26^, Guangxi^27^ and other provincial and prefecture (city) scales as well as the Yangtze River basin^28^, the Yellow River Basin^29^, and other cross-provincial regions as supplementary subhotspots, while the survival of minority villages in the Southwest with Guizhou as the centre of gravity has received less attention and the formation mechanism is not thorough.

Given that existing studies lack deep-level micro variable explanations for traditional villages, we explored the intrinsic correlations and characterised the spatial characteristics of natural factors and human factors, which influence the geographic pattern of traditional villages. The progress relied on GIS, spatial process and mechanism analysis, geographic probes, and other technical means and theoretical methods. Compared to previous studies, we emphasised the quantitative analysis of deep-seated influencing factors and their driving forces on the distribution pattern of traditional villages. The analysis results will help people further understand the limiting factors and driving mechanisms for the long-term maintenance of traditional villages, and provide a new research perspective for the protection and sustainable utilisation of traditional villages in the mountainous areas of China and other countries worldwide.

## Study area

**Figure 1:**
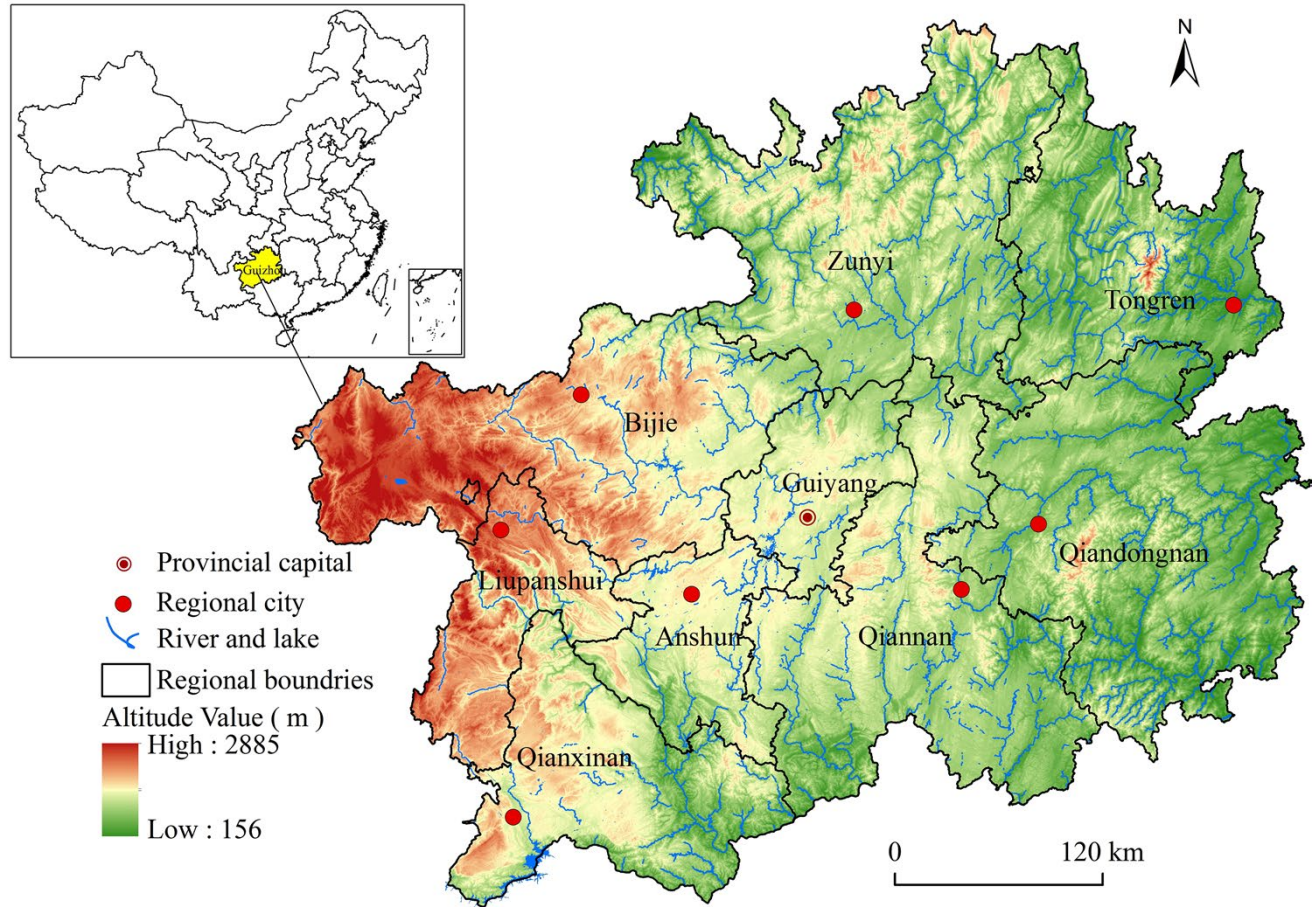
Map of the study subjects in China. Maps were created using ArcGIS10.2 (Environmental Systems Research Institute, USA. https://www.esri.com/).

We used Guizhou as the research area (Fig. 1). Guizhou is in the Yunnan-Guizhou Plateau in Southwest China, is a transitional slope zone from east Yunnan to Sichuan, Hunan and Guangxi, is a watershed area of the Yangtze River and the Pearl River systems, and is located at latitudes of 24°37′–29°13′ N and longitudes of 103°36′–109°35′ E. It covers an area of 176,400 km^2^ and has an average elevation of approximately 1100 m, in which the average annual precipitation is 1000–1400 mm, the climate type belongs to the subtropics, and various topographic types. Moreover, it has 18 ethnic groups living for generations that have diverse ethnic cultures and is one of the regions with the largest number of traditional villages in China.

## Data source and method

### Data source

In this paper, 724 traditional villages in Guizhou, which were evaluated and recognised by the Ministry of Housing and Urban‒Rural Development and other units as of 2019, were taken as the research subjects, and the corresponding spatial coordinates were obtained, converted, and projected through the search function of Baidu Maps. The data of the administrative division of the province, 1:500,000 DEM, hydrogeology, climate, and meteorology, came from http://gre.geodata.cn/ and http://www.karstdata.cn/. The land use, vegetation (*NDVI*) and traffic data came from http://www.dsac.cn/. Some economic and social data were sourced from the Guizhou Statistical Yearbook 2019. The other data were generated by us.

## Research methods

### Average nearest neighbour analysis

In this paper, we mainly measured the distance between a traditional village and its nearest neighbour traditional village, calculated its average value^30^, and finally obtained the nearest neighbour index *R* value that can explained the aggregation or dispersion of spatial data, which is expressed as follows.

1 $$R=\frac{{\bar{D}}_{o}}{\mathop{D_E}\limits_{}^{--}}=(\frac{1}{n}\sum_{i=1}^{n}{d}_{i})/(\frac{1}{2\sqrt{n/A}})$$

where ${\bar{D}}_{o}$ is the observable average distance between a traditional village and its nearest neighbouring traditional village, *D*_*E*_ is the expected average distance between a traditional village and a given random pattern, *n* is the number of traditional villages, *d*_*i*_ is the distance between traditional village *i* and its nearest neighbouring traditional village within the province, and *A* is the area of the region constituted by the spatial coordinates of the outermost traditional village. When *R* > 1, it is uniformly distributed; when *R* = 1, it is randomly distributed; and when *R* < 1, it is aggregated.

### Tyson polygon analysis

The Tyson polygon analysis method was initially proposed by A.H. Tyson, a Dutch climatologist, who used a regional blockisation method based on discrete distribution points^31^. This method uses the coefficient of variation in the Tyson polygon area for calculation. The coefficient of variation can be defined as the ratio between the standard deviation of the Voronoi polygon area of a traditional village and its average value. Here, it could be used to measure the relative degree of change in the spatial distribution of traditional villages and is calculated as follows:

2 $${\text{C}}{\text{V}}= \text{ S/M}$$

In the formula, *S* is the standard deviation of the Voronoi polygon area, and *M* is the average value of the Voronoi polygon area.

### Nuclear density analysis

The method can better reflect the scope and impact of a certain thing on the surrounding area. In this paper, it reflected the distribution hotspots and overall pattern of traditional villages^32^, specifically in terms of the probability of traditional villages occurring in areas with high density and vice versa with low probability^33^. The calculation is as follows:

3 $${f}_{n}\left(x\right)=\frac{1}{nh}\sum_{i=1}^{n}k\left(\frac{x-{x}_{i}}{h}\right)$$

Here, *k* is the weight function of the kernel; *n* is the number of traditional villages; *h* is the bandwidth, which is the width that the surface with *x*(traditional villages) as the origin extends in space, and the value of *h* will affect the smoothness of the graph; and *x*-*x*_*i*_ is the distance between the density estimation points *x* and *x*_*i*_.

### Spatial process and mechanism analysis

**Figure 2:**
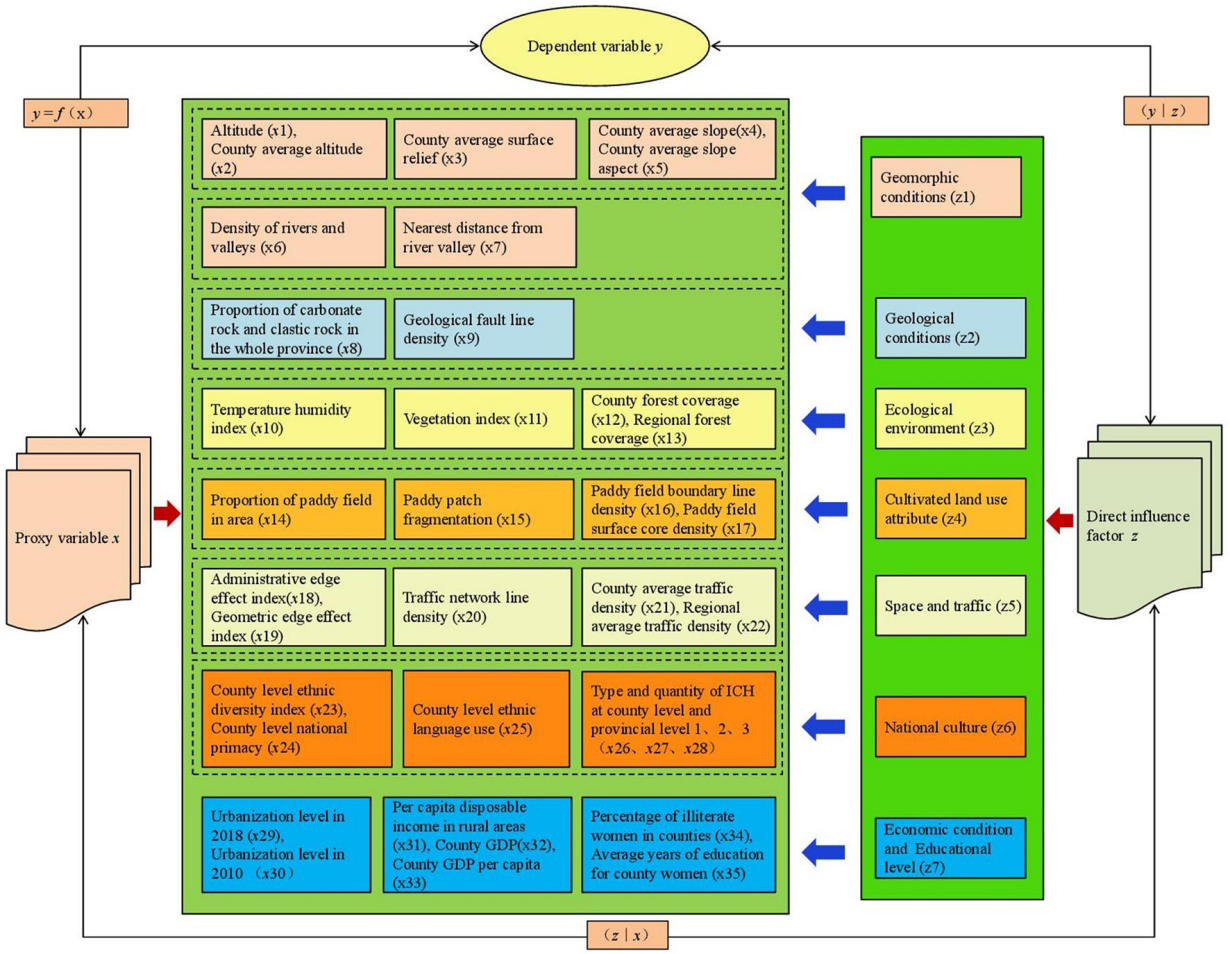
Proxy variable diagram for spatial process and mechanism analysis.

Spatial processes and mechanisms are expressed in terms of the linkage (*y*|*z*) between the spatial distribution density of traditional villages (dependent variable *y*) and natural and socioeconomic factors (direct influence factor *z*). In the process of geographic spatial analysis, however, the influence factor *z* is generally difficult to obtain directly, and it is usually necessary to find the proxy variable *x* that is as correlated as possible with the direct influence factor *z* (*z*|*x*) and covers the study area and then construct the dependent and proxy variables. The statistical relationship between the dependent and proxy variables is *y* = *f* (*x*)^34^, as shown in Fig. 2.

In this paper, the influence factors *z* closely related to traditional village distribution included geomorphic conditions, geological conditions, ecological environment, cultivated land use attribute, space and traffic, national culture, economic condition and educational level. These influence factors covered 35 proxy variables, which were derived from attributes for influence factors of nature and humans.

### Ethnic diversity analysis

Worldwide, areas with high biodiversity are often inhabited by ethnic minorities, and cultural diversity is also a highly importantarea^35^. The ethnic diversity index mainly adopts the biological Simpson index method, which can measure the level of biological population, and the higher the index indicates good community diversity, as follows:

4 $$D=1-\sum {P}_{i}^{2}$$

In the formula, *D* represents the ethnic diversity index in the county area of Guizhou Province, and *P*_*i*_ represents the proportion of the *i*th ethnic population to the total number of ethnic populations in the county area.

The primacy index reflects the concentration of the largest groups in the region. In this paper, we chose the largest and second largest ethnic population in each county area of Guizhou Province as the ethnic primacy index.

5 $$S={P}_{1}/{P}_{2}$$

where *S* is the ethnic primacy index, *P*_1_ is the largest ethnic population in each county area, and *P*_2_ is the second largest ethnic population in each county area.

The degree of ethnic language use reflects the condition of ethnic languages used in the county area of Guizhou Province, which was classified as five categories: not used, used only sporadically by elderly individuals, used only sporadically by the middle-aged, used sporadically by elderly individuals, middle-aged, young and childhood, and used frequently by elderly individuals, middle-aged, young and childhood.

### Geographic detector analysis

The spatial variation of a certain independent variable *z* and its degree of consistency are expressed by detecting to what extent a certain independent variable *z* explains the dependent variable *y*, which is expressed by the *q* value. In this paper, ArcGIS was used to spatially overlay the traditional village distribution layer with each driver layer and obtain the value of each type variable *z* that directly affected the driver through reclassification. The dependent variable *y* was a numerical variable that expressed the density attribute of the spatial distribution of traditional villages in the province, the independent variables *z*1 to *z*7 were type variables, including geomorphology, geology, ecology, land use, transportation, ethnic culture, and economic condition, education level, etc., and the proxy variable *x*, which was associated with these variables, was the categorical attribute value. The *q* values were obtained by substituting the *y* and *x* data into the Geodetector software run, and its value range was [0, 1], and the larger the value, the stronger the explanation of the dependent variable y by the independent variable factor *z*. The model is as follows^36^.

$$q=1-\frac{\sum_{h=1}^{L}{N}_{h}{\sigma }_{h}^{2}}{N{\sigma }^{2}}=1-\frac{SSW}{SST}$$

6 $$SSW=\sum_{h=1}^{L}{N}_{h}{\sigma }_{h}^{2}, SST=N{\sigma }^{2}$$

where *h* = 1, …, *L* is the classification of dependent variable *y* or independent variable *z*; *N*_*h*_ and *N* are the number of cells in layer *h* and the whole area, respectively, and are the variance of *y* values in layer *h* and the whole area, respectively; and *SSW* and *SST* are the sum of variance within the layer and the total variance in the whole area, respectively.

## Results

### Distribution pattern of traditional villages

#### The geographical spatial distribution of traditional villages

**Figure 3:**
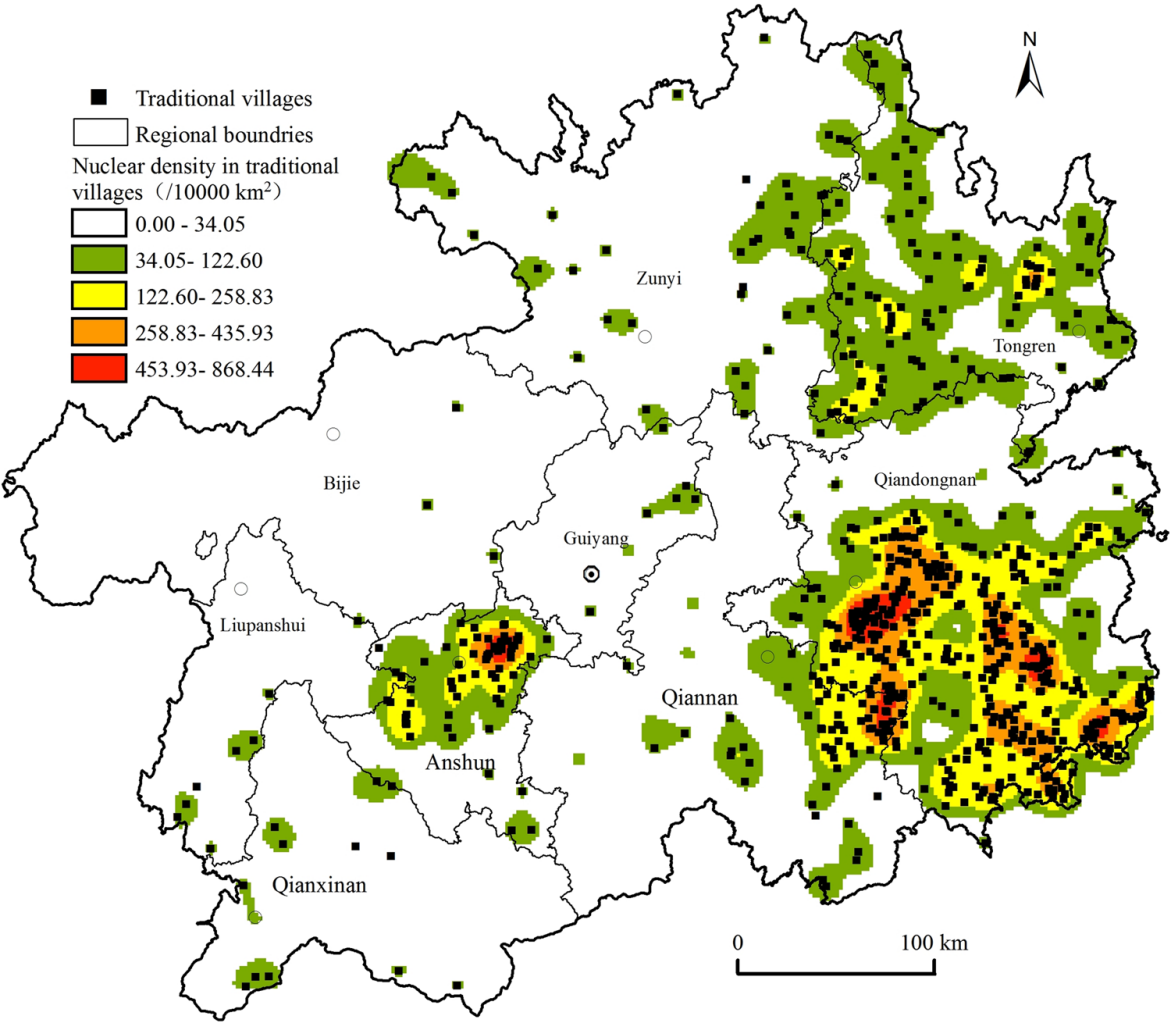
Spatial distribution of nuclear density in traditional villages in Guizhou. Maps were created using ArcGIS10.2 (Environmental Systems Research Institute, USA. https://www.esri.com/).

Taking 724 traditional villages in Guizhou as the research subjects, the observable mean distance (‾*D*_*o*_) of 5325 m and the desired mean distance (‾*D*_*E*_) of 8804 m were obtained based on the nearest neighbour analysis model, and their nearest neighbour index *R* was 0.60, indicating that the traditional villages in the province showed high clustering characteristics. To further verify the nearest neighbour index of traditional villages in the province, Tyson polygon statistical analysis of traditional villages based on ArcGIS software was conducted, and the standard deviation values and mean values were obtained as *S* = 625796067 m^2^ and *M* = 244129223 m^2^, and the coefficient of variation *C*_*V*_ was 2.56 (≥ 64%)^37^, which determined the traditional villages in Guizhou as cluster distribution (Fig. 3). Overall, high clustering with very high primacy was found in Leishan, Liping, Congjiang, Taijiang, Rongjiang, Jianhe, Qiandongnan and Sandu of Qiannan, as well as Xixiu and Pingba of Anshun, and extended to Shiqian, Sinan, and Dejiang of Tongren in northeastern Guizhou, as well as Fenggang and Wuchuan of Zunyi at the junction, accumulating more than 60% of the total, while other areas were scattered.

#### The edge effect of traditional villages

**Figure 4:**
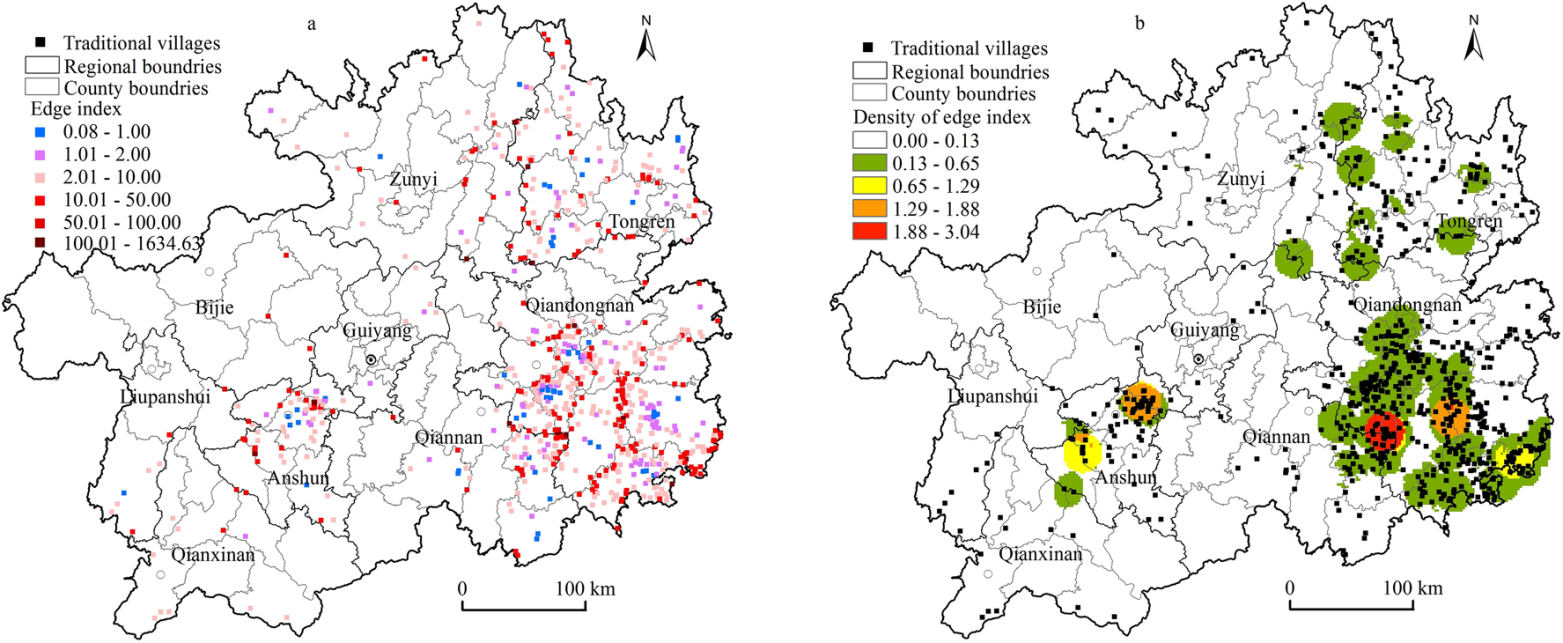
Spatial distribution of the edge index and its density in traditional villages in Guizhou. Maps were created using ArcGIS10.2 (Environmental Systems Research Institute, USA. https://www.esri.com/).

Administrative boundary areas often have the distinctive feature of interlacing multiple cultural and economic forms, creating objective conditions for the siting and retention of traditional villages^10^, and previous scholars have measured the distance between traditional villages and central towns (cities) as the spatial relationship between the two^38^; however, this is not sufficient to express the edge effect qualities between them. There is a lack of further quantification and explanation of how the two variables of the distance between villages and central towns (municipalities) and between villages and regional administrative boundaries jointly affect this. In this paper, the ratio *D* of the distance *d*_1_ from the village point to the county administrative centre of the jurisdiction to the distance *d*_2_ from the village point to the nearest administrative boundary within the jurisdiction (county) was used to express its edge effect. When the distance *d*_1_ from the traditional village to the county administrative centre was greater than the distance *d*_2_ from the nearest county boundary, i.e., the index *D* > 1, it indicated that the traditional village had an edge effect, and the larger its value was, the more obvious the edge effect; when the index *D* = 1, then it indicated that the edge effect was not obvious; if *D* < 1, it indicated that the traditional village was adjacent to the county administrative centre and was subject to greater intervention by the relatively developed socioeconomic level and had no edge effect. The results of the study showed that the number of traditional villages without edge effects or that were not obvious (*D* ≤ 1) was 54, accounting for 7.46% of the total; 670 traditional villages with edge effects (*D* > 1) accounted for 92.54% of the total. More than 90% of the traditional villages in the province showed significant edge effects (Fig. 4a), such as the traditional village of the Hepian Group in Huaqiu Town, Tongzi County, and Zunyi City, which was known as "one step across three counties". In addition, there were obvious hot spots of edge effects at the junction of adjacent counties, regions, and provinces, such as Leishan-Danzhai-Rongjiang and Sandu in Qiannan, Liping and Rongjiang in Qiannan, Pingba and Xixiu, Zhenning and Guanling in Anshun, and Liping-Conjiang and Sanjiang in Guangxi in Qiannan and northeast Guizhou, which had the distinctive feature of crossing the boundaries of 2–4 county administrative units (Fig. 4b), as seen in Fig. 3. The areas with significant edge effects had high spatial consistency with the high-density distribution areas of traditional villages.

### Spatial distribution of different natural factors in traditional villages

#### Distribution in elevation and longitude

**Figure 5:**
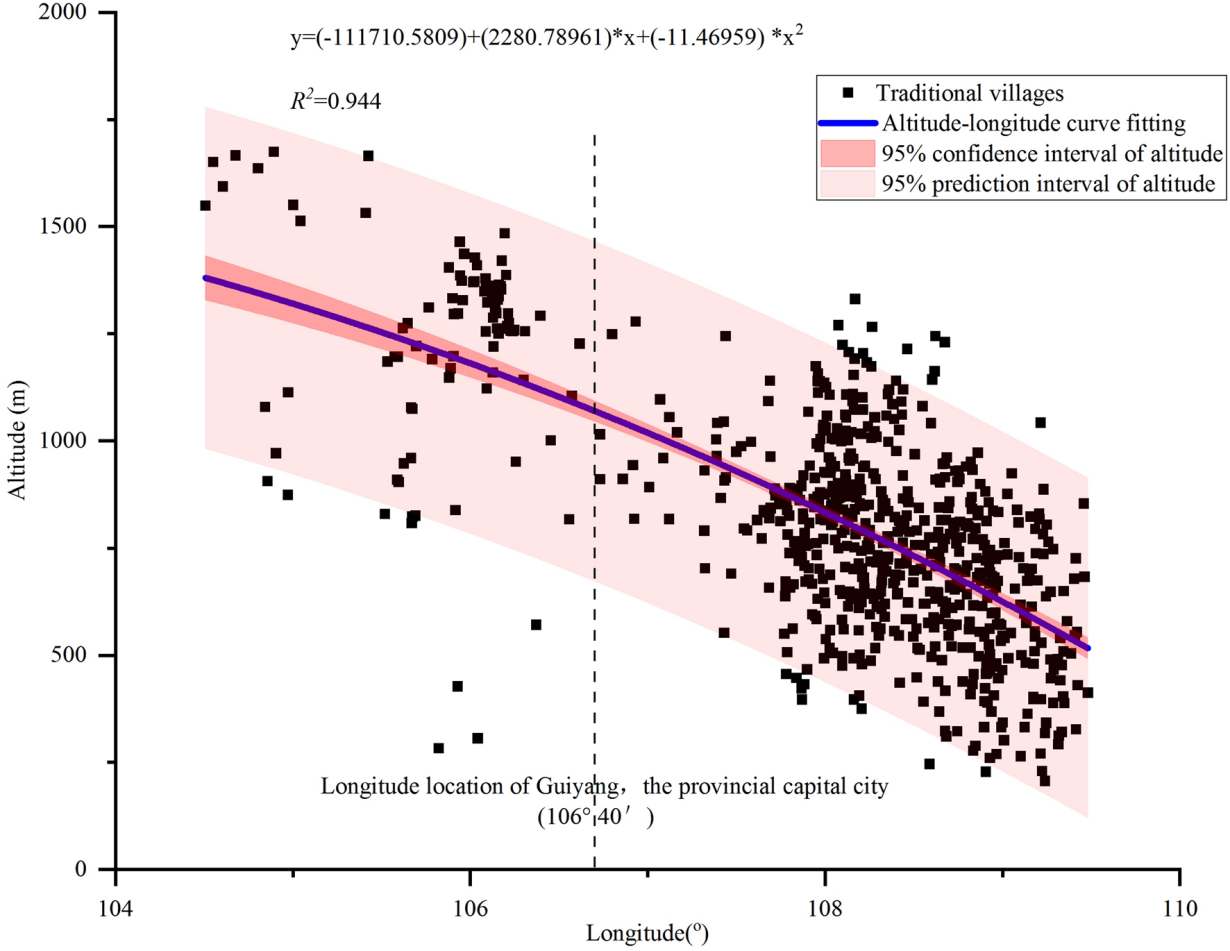
Spatial distribution of altitude and longitude of traditional villages in Guizhou.

Through the statistical analysis of the altitude of traditional villages, their skewness and kurtosis were0.490 and 0.136, respectively, which showed that the traditional villages in the province had an approximately normal distribution with left skewness in the altitude vertical direction. The spatial variation was as follows: ① 710 traditional villages were distributed in the altitude range of 250–1500 m, accounting for 98.07% of the total number, among which the proportion of 750–1000 m was the largest, with 252 villages (Table 1). ② The distribution density of villages gradually decreased with increasing altitude, and the maximum density was ≤ 500 m altitude area. ③ The traditional villages in the east, with Guiyang, the capital of the province, as the dividing line, were concentrated at 250–1250 m (Fig. 5), and in the west, they were concentrated at 800–1700 m, all showing the characteristics of being far from the central city. ④ The altitude of traditional villages and their longitude were spatially significantly negatively correlated, with a Pearson correlation coefficient of 0.68 and a curve fit of 0.944, and both passed the *F* test and *T* test, indicating statistical reliability, while there was no correlation with their latitude. Furthermore, traditional villages in the province were generally spread along the longitude direction and showed a pattern of "dense in the east and sparse in the west", mostly distributed in the low-altitude slope areas in the transition from the Yunnan-Guizhou Plateau to Chongqing, Hunan, and Guangxi. This may be related to the overall east‒west trend of river basins in the province, which in turn influences the evolution and distribution of regional farming civilisation settlements.

**Table 1:** Elevation classification and distribution of traditional villages in Guizhou.

Altitude (m)	Area (km^2^)	Area ratio (%)	Numberoftraditional villages	Proportion of traditional villages at different elevations	Density of traditional villages (/10,000 km^2^)
≤ 500	8832.68	5.01	90	12.43	102
500–750	27,891.58	15.83	223	30.80	80
750–1000	44,317.11	25.16	252	34.81	57
1000–1250	37,289.48	21.17	96	13.26	26
1250–1500	28,531.83	16.20	53	7.32	19
1500–1750	12,468.96	7.08	10	1.38	8
> 1750	16,835.36	9.55	0	0	0
Total	176,167.00	100.00	724	100.00	45

### Distribution in surface relief degree

Using the neighbourhood analysis tool of ArcGIS, the surface undulation degree based on DEM data of the province was calculated and spatially overlaid with traditional villages. The results showed that the average surface relief of traditional villages in the province was 75.44, which was higher than the average value of 69.00 for 213,885 natural villages in the province, and the surface relief of traditional villages in nonkarst areas was 79.64, while in karst areas, it was 67.10. This further indicated that traditional villages were mostly distributed in mountainous areas with large relief in nonkarst areas, while traditional villages in karst areas were located on flatter terrain. This was evidenced by the Miao hanging foot tower villages in Qiandongnan in nonkarst areas and Tunbao villages in Puding County in karst areas; the surface relief of traditional villages in Qiandongnan and Qiandongnan was the highest, at 87.61 and 79.75, respectively, followed by Liupanshui, Qianxinan, Bijie, and Tongren, while Guiyang and Anshun were the lowest, at 60.29 and 47.58, respectively.

#### Distribution in gradient and aspect

① Distribution on different slopes. The slope distribution range of traditional villages in the province extracted from DEM data was 0.39°–45.25°, 23 slope intervals and the corresponding number of traditional villages were obtained by grading at an interval of 2°, the skewness was calculated to be 0.41 and the kurtosis was −1.28, and by normal Q‒Q test, the traditional villages in the province showed an approximately normal distribution with slope; among them, 8°–24° slope concentrated. The average slope of traditional villages was 15.98°, which was greater than the average slope of natural villages in the province of 13.68°, different from Zhejiang traditional villages, which were mostly distributed in areas with smaller slopes; it could be seen that Guizhou showed obvious spatial differentiation on different slopes and was mostly located in mountainous areas with larger slopes. (2) Distribution on different slopes. Using the "surface analysis" function of ArcGIS, the slope map of the province was obtained from DEM data, which mostly included the northern slope (0–22.5°, 337.5°–360°), northeastern slope (22.5°–67.5°), eastern slope (67.5°–112.5°), southeastern slope (112.5°–157.5°), south slope (157.5°–202.5°), southwest slope (202.5°–247.5°), west slope (247.5°–292.5°), and northwest slope (292.5°–337.5°), and extracted the slope direction values of traditional village locations by spatial superposition analysis. It was found that the number of traditional villages with southeast slope and east slope orientation was the largest, accounting for 17.82% and 16.30%, respectively, and the least was the north slope, accounting for only 3.87%; if divided by two types of slopes, the yang slope (90°–270°) and the yin slope (0–90°, 270°–360°), the number of traditional villages in the province was 429 for the yang slope. The number of traditional villages in the province was 429 on sunny slopes and 295 on shady slopes, with a distribution ratio of 1.45:1, and their sunny characteristics were obvious. We also found that the ratio of sunny slopes to shady slopes in traditional villages in karst areas was 1.81:1, while in nonkarst areas, it was 1.31:1, which showed that the location of traditional villages in karst areas requires more sunny slope attributes, while in nonkarst areas, due to the wider spatial width suitable for survival, the selection of slope orientation was less strict than the former.

#### Distribution in lithology, fault line, and river system

① Distribution in lithology. The exposed area of carbonate rock in Guizhou accounted for 61.90% of the total area of the province, while the distribution of clastic rock accounted for only 38.10%; the results showed that 482 traditional villages were distributed in clastic rock areas, accounting for 66.57% of the number of traditional villages, while carbonate rock areas accounted for only 33.43%, and their overall spatial distribution characteristics were opposite to the lithological distribution area, which was related to the fact that clastic rock areas had more convenient utilisation of the spatial distribution pattern of the two areas was also significantly different, as shown by the nearest neighbour index R and coefficient of variation of 0.68 and 1.64 for traditional villages in the carbonate area and 0.49 and 3.64 in the clastic rock area, respectively. Thus, it could be seen that both lithologies were clustered, but the clastic rock area showed greater clustering and distribution along the fault line. ② Distribution along the fault lines. Based on 13,852 fault lines in the province, four buffer zones of 500–2000 m were constructed and spatially overlaid with 724 traditional village sites in the province. The results showed that more than half of the traditional villages were distributed within 1500 m of the fault lines, accounting for 53.87%, and they were mostly distributed in a northeast‒southwest fault extension, which had a high consistency with the spatial distribution of spring sites in the province, further indicating that fault lines controlled the formation and evolution of water sources and intermountain dam areas and indirectly influenced the humanistic process of traditional villages in terms of site selection and farming. ③ Distribution on river systems. The distance from traditional villages to the nearest rivers was measured by ArcGIS proximity function analysis, and it was found that the average length of traditional villages to the nearest rivers (or streams) was 480 m, showing obvious proximity characteristics. However, there were differences in the proximity distance between karst and nonkarst areas; specifically, the average distance of traditional villages to the nearest rivers was 596 m in karst areas and 422 m in nonkarst areas. The main reason for this was that the density of surface water systems in karst areas was smaller than that of nonkarst areas in the same climatic and biological environment. In terms of spatial distribution with water sources (spring points), the average distance from traditional villages to the nearest water source in the province was2980 m, among which the average distance of traditional villages distributed in karst areas was2357 m from the nearest water source, which was approximately 1000 m closer than the distance from traditional villages to the nearest water source in nonkarst areas. This was related to the clustered distribution of water resources along a water barrier or fracture zone in karst areas, resulting in traditional villages in this area having the characteristics of spreading near karst springs, while nonkarst areas had more streams or mountain springs as their water sources were denser and could be diverted down through ditches or pipe networks, which were less restricted by water source types and terrain than the former, and each was small. This provided a relatively independent ecological space for the survival of local villagers, which was more conducive to the continuity and development of villages.

#### Distribution of the temperature-humidity index

**Figure 6:**
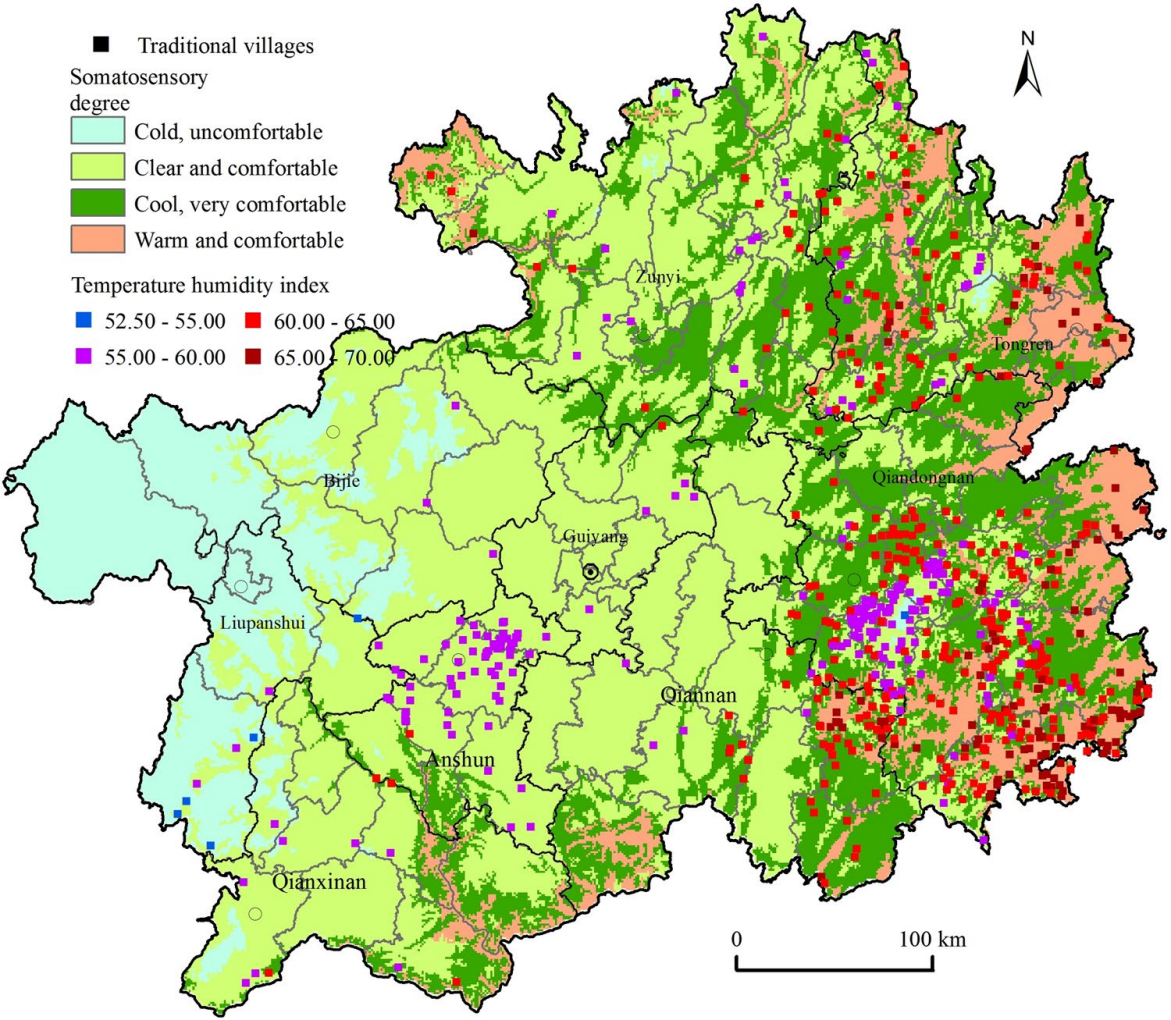
Spatial distribution of traditional villages and temperature humidity index in Guizhou. Maps were created using ArcGIS10.2 (Environmental Systems Research Institute, USA. https://www.esri.com/).

Climatic conditions are an important factor limiting population distribution, and suitable climatic resources are crucial for sustainable human reproduction and development. We used the annual average temperature and humidity-related data of the province, obtained the temperature and humidity raster data of the province with the help of the ArcGIS spatial interpolation function, drew the spatial distribution of the temperature-humidity index of the province by referring to the temperature-humidity index model^39^, and extracted the corresponding temperature-humidity index image element values of traditional villages (Fig. 6). The temperature-humidity indices of traditional village locations in the province ranged from 52.50–65.47, with comfortable and very comfortable grades accounting for 99.17% of the total and the colder and more uncomfortable somatic sensations accounting for only 0.83%. According to the temperature-humidity index physioclimatic grading standard^40^, traditional villages in Guizhou were mostly cool and comfortable somatic environments (Table 2), which were high-quality resources for the continuous healthy development of village residents from the scale of the province. Traditional villages with very comfortable physical sensation were mostly distributed in Taijiang, Jianhe, Liping, Congjiang, and Rongjiang of Qiandongnan, and in Sandu, Libo of Qiannan, and in Shiqian, Sinan of Tongren, while traditional villages with comfortable physical sensation werecluster spaces constituted by the core of Leishan and Jianhe in Qiandongnan, Xixiu and Pingba in Anshun, and the border between Liping, Congjiang in Qiandongnan and Sanjiang in Guangxi Province, while other areas were scattered.

**Table 2:** Temperature humidity index and quantity distribution of traditional villages.

Temperature humidity index (THI)		Number of traditional villages (nos.)	Proportion (%)
Range value	Somatosensory degree		
< 40	Extremely cold and uncomfortable	0	0
40–45	Cold and uncomfortable	0	0
45–55	Cold, uncomfortable	6	0.83
55–60	Clear and comfortable	239	33.01
60–65	Cool, very comfortable	357	49.31
65–70	Warm and comfortable	122	16.85
70–75	Slightly hot, more comfortable	0	0
75–80	Sultry and uncomfortable	0	0
> 80	Extremely sultry and uncomfortable	0	0

#### Distribution of vegetation coverage

Areas with high vegetation cover tend to have higher levels of biodiversity, and the reciprocal feeding mechanism between biodiversity and traditional culture is obvious^41^. As traditional villages are the carriers of traditional culture and vernacular knowledge inheritance^42^, is there some intrinsic correlation between vegetation cover and traditional villages as well?

**Figure 7:**
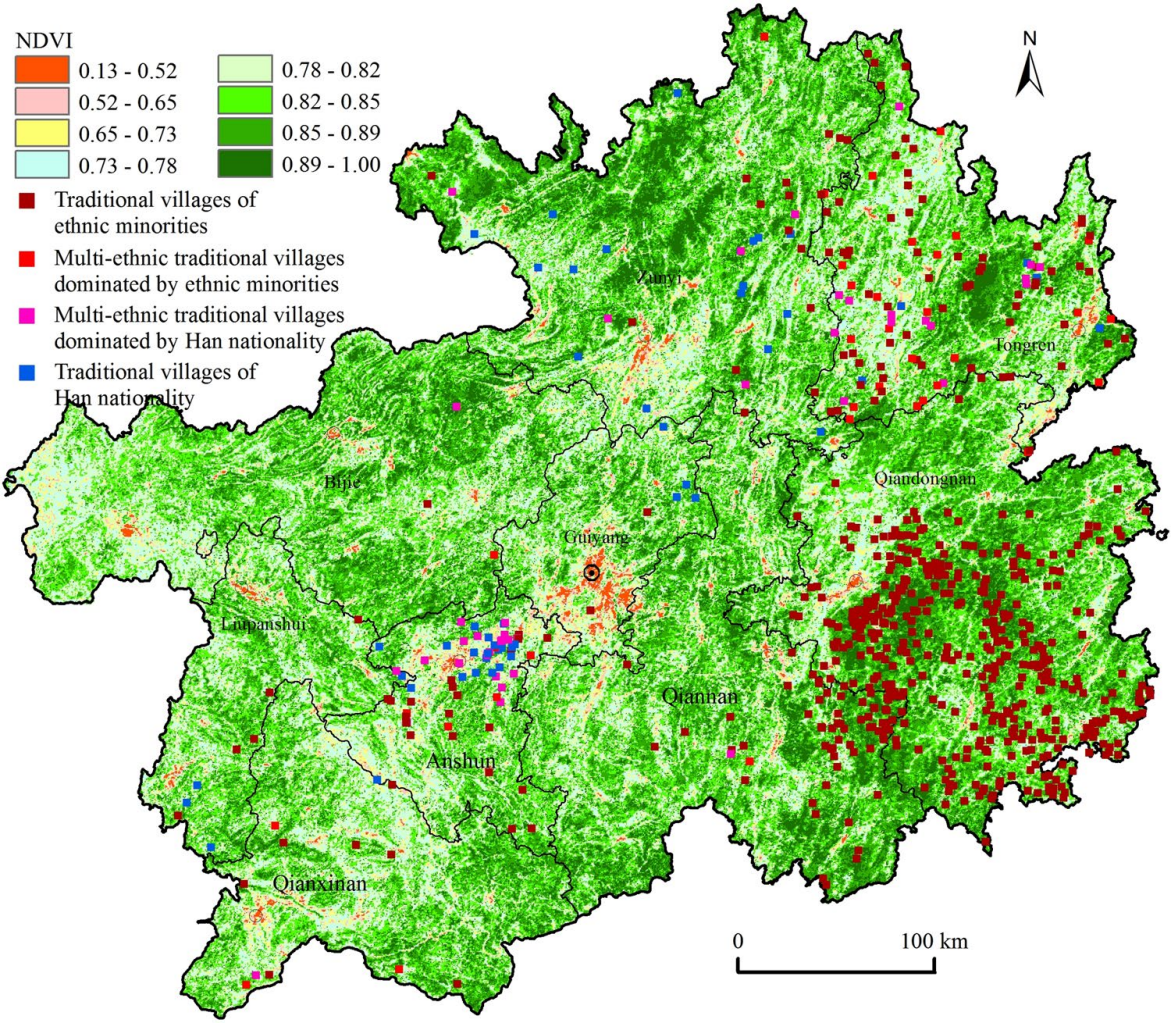
Distribution of traditional villages and vegetation index in Guizhou. Maps were created using ArcGIS10.2 (Environmental Systems Research Institute, USA.  https://www.esri.com/).

To answer this, we conducted a spatial overlay using traditional villages and the normalised vegetation index (*NDVI*) in 2016 and found that traditional villages were mostly distributed in areas with a high vegetation index, mostly involving the regions of Qiandongnan, Tongren, Zunyi, and Qiannan, but there was no lack of spatial heterogeneity of traditional villages such as Xixiu and Pingba of Anshun, which were highly clustered and located in areas with a low vegetation index. Furthermore, the average vegetation index of traditional villages was 0.79, which was higher than the provincial average vegetation index of 0.66. The high vegetation coverage around traditional villages reflected the respect and protection of the ecological environment by the residents of traditional villages, and on the other hand, the good ecological environment further maintained the structural stability and functional diversity of the ecosystem around the villages, thus promoting and guaranteeing the sustainable development of traditional villages. Due to land fragmentation, frequent soil erosion, a high degree of rock desertification, and a lack of surface water resources in karst areas, the overall level of vegetation cover was low, and the output capacity per unit of land was weak. Thus, it was difficult to support more people, and the village landscape was small and scattered, causing it to be difficult to form a high-density gathering. The nonkarst area had strong contiguous land, good water and heat conditions, and high vegetation cover, which could provide more material and energy per unit area than the former, resulting in a high concentration of villages. In terms of the ethnic attributes of traditional villages in Guizhou, traditional villages in ethnic minority areas were mainly distributed in areas with better vegetation conditions, while traditional villages dominated by Han Chinese were mainly distributed in mountainous flat dam areas with average vegetation cover but better agricultural conditions (Fig. 7).

### Spatial distribution of different humanistic and social factors in traditional villages

#### Distribution in ethnic diversity index, ethnic primacy index, degree of ethnic languages used, and types and quantities of ICH

**Figure 8:**
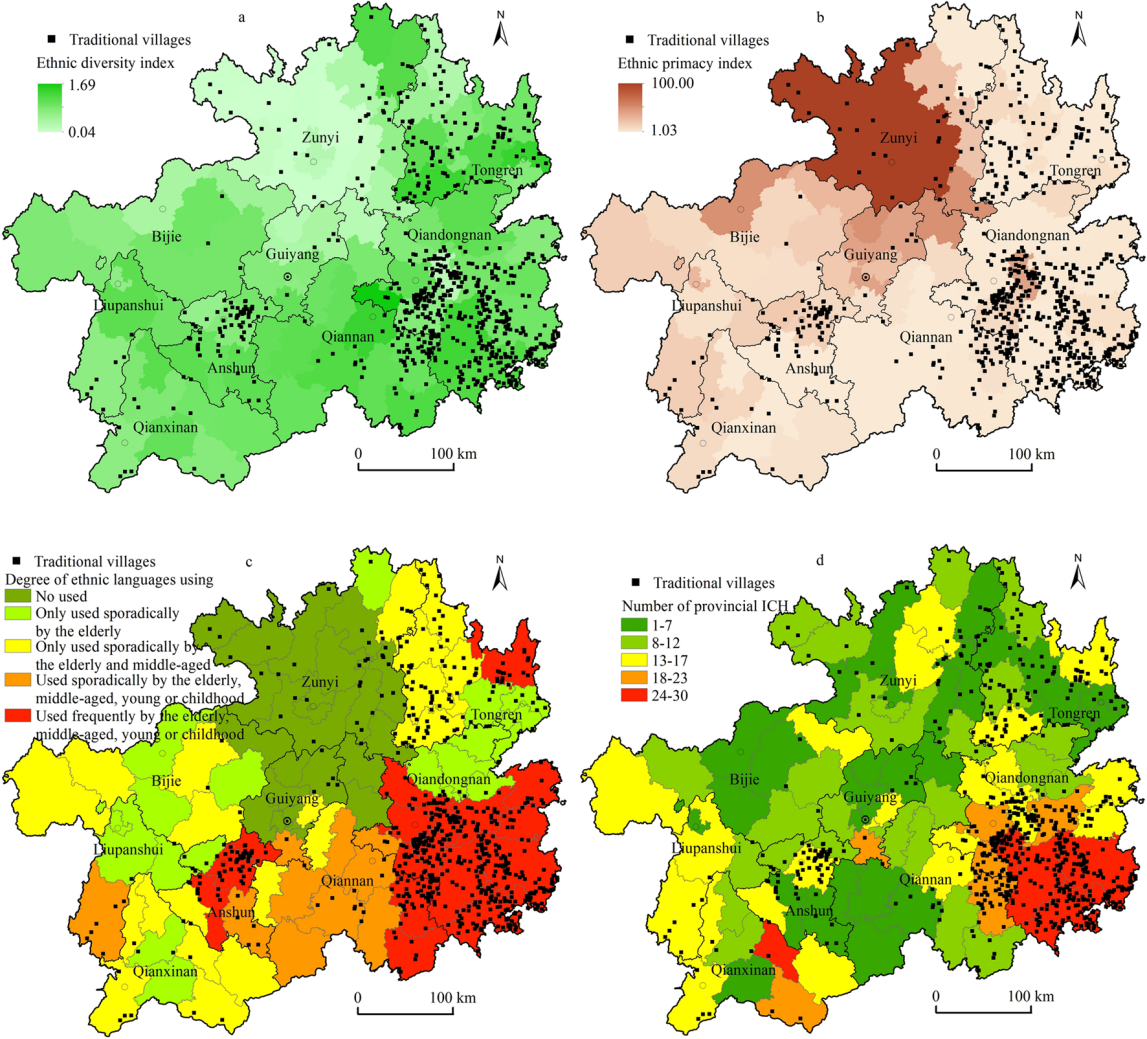
Distribution of traditional villages and ethnic cultural diversity in Guizhou. Maps were created using ArcGIS10.2 (Environmental Systems Research Institute, USA. https://www.esri.com/).

The spatial correlations existing between traditional villages and different ethnic cultural attributes in the region were further explored by measuring the county ethnic diversity index, ethnic primacy, ethnic language usage level, and the type and number of ICHs. The results showed that the regions with high ethnic diversity levels in the province were mostly located in the local areas of Qiandongnan, Qiannan, and Tongren, in addition to Zunyi and Guiyang (Fig. 8a), which were mostly the gathering places of Miao, Dong, Buyi, Shui, Tujia, and Gelao ethnic minorities and were also the core areas of traditional village distribution. We also found high-density clusters of traditional villages in Leishan and Taijiang in Qiandongnan and Anshun. This was related to the relatively homogeneous types of ethnic minorities in the region. As shown in Fig. 8b, except for the positive correlation between traditional villages and ethnic primacy in Taijiang and Qiandongnan, other regions generally showed a negative correlation between the distribution of traditional villages and ethnic primacy. We constructed a spatial distribution map of the degree of minority language use based on county units from the survey of minority language use. Figure 8c shows that traditional villages had a high spatial consistency with the degree of minority language use, especially in the large part of Qiandongnan where high-density traditional villages were distributed, and in the spatial consistency between traditional villages and where the degree of minority language use was high, especially in the large part of Qiandongnan, Sandu of Qiannan, and Pingba, Xixiu of Anshun where high-density traditional villages were distributed. As a product of long-term accumulation and continuation of a region, ICH was mostly attached to carriers such as ancient towns, ethnic villages, and traditional villages. As of 2019, Guizhou has announced five batches of provincial-level ICH with a total of 713 items and 827 sites. Moreover, we constructed a spatial distribution map of ICH resources in each district (county and city) with the county as a unit and spatially overlays it with traditional villages. We found that, except for the high density of traditional villages and low number of ICHs in northeast Guizhou, Pingba and Xixiu of Anshun, the Qiandongnan area showed a significant positive correlation between the distribution of traditional villages and the number of ICHs (Fig. 8d).

### Distribution of the attributes of cultivated land use

**Figure 9:**
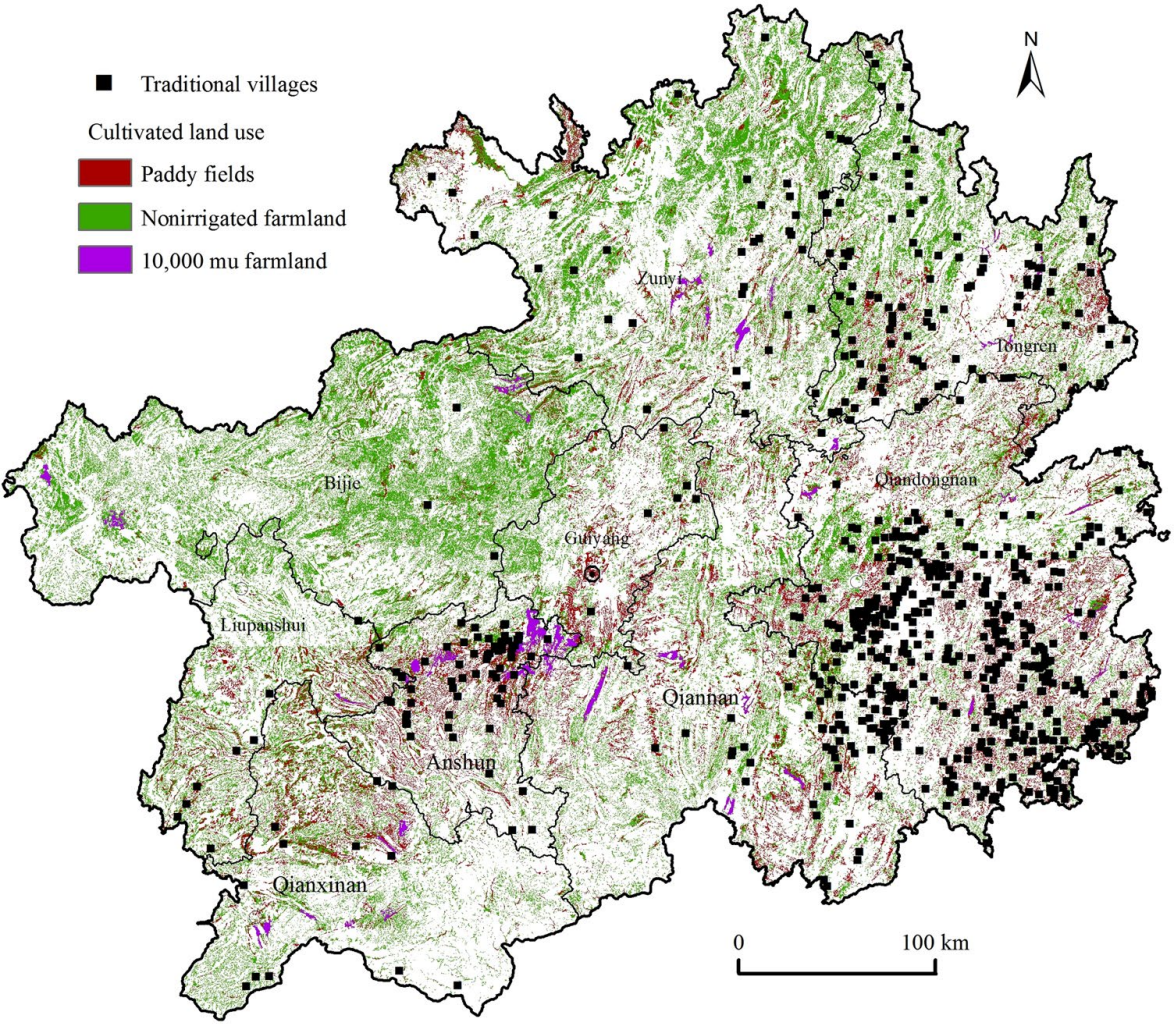
Spatial distribution of traditional villages and cultivated land. Maps were created using ArcGIS10.2 (Environmental Systems Research Institute, USA.  https://www.esri.com/).

Two types of arable land, paddy fields, and dry lands, as well as the more concentrated and contiguous 10,000 mu dam area, were selected to characterise the distribution of arable land in the province and was spatially overlaid with traditional villages (Fig. 9). In general, traditional villages in Guizhou weremostly distributed in the counties and cities of Qiandongnan, Anshun, Tongren, and Qiannan, where there were more arable lands, especially paddy fields, unlike traditional villages in Hunan, which were far from arable lands^38^; it was found that these traditional villages had the distinctive feature of being distributed near paddy fields (Fig. 9), and their inhabitants maintained "rice-fish symbiosis" and "rice-duck symbiosis", which enables them to achieve the purpose of "one land with two uses, one water with two uses, and double or multiple harvests in one season"^43^. The yields were greatly increased, such as in the case of the "traditional rice, fish and duck symbiotic agricultural production model—rice, fish and duck system in Congjiang Dong Township, Guizhou", which has supported the villagers to live and work happily for generations and to expand^19^, fully reflecting the special function of rice fields to carry traditional civilisation continuously. It was estimated that the nearest distance of traditional villages from the 10,000 mu farmland was at least 6 km, and the larger distance was tens to hundreds of kilometres, which had the spatial attribute of being far from the high quality arable land resources such as the mountain flat cultivated land, especially the 10,000 mu farmland.

#### Distribution in the traffic road network

Based on the unit of prefectures (cities), the road mileage of each region was obtained by superimposing the road network data above the township level, the traffic road network density of the province was obtained by the ratio of road mileage to the area of prefectures (cities) administrative districts, and the distribution of traffic road network and traditional village density in each prefecture (city) was extracted as shown in Table 3. The results showed that the distribution of traditional villages in the province and the level of traffic development are of the following types: first, places (states and cities) with developed traffic but the low density of traditional village distribution, mainly represented by Guiyang and Liupanshui areas, have the characteristics of good geographical location conditions or high road network density; second, areas with more developed traffic and larger traditional village distribution, such as Anshun, Tongren and Zunyi areas; third, areas with less developed traffic and low density of traditional village distribution; and fourth, areas with relatively poor transportation conditions but a high density of traditional village distribution, which is mainly represented by the Qiannan and Qiandongnan areas, and is the main area of traditional village distribution in the province.

**Table 3:** Density distribution of traffic network and traditional villages in each prefecture (city) of Guizhou.

Region	Traffic network density (km/km^2^)	Density of traditional villages (piece/10,000 km^2^)
Guiyang	0.81	8.71
Liupanshui	0.40	10.09
Anshun	0.38	72.07
Tongren	0.31	61.10
Zunyi	0.31	12.68
Qianxinan	0.30	6.55
Bijie	0.28	1.12
Qiannan	0.25	26.34
Qiandongnan	0.23	134.48

#### Distribution at the urbanisation level

The urbanisation rate is one of the important indicators to measure the modernisation process of a country and region, and the urbanisation process is to some extent characterised by obvious untraditional cultural and customary attributes. The urbanisation rate of the household population in counties in 2018 was taken as an indicator and expressed through the spatial overlay of traditional villages and urbanisation level. The study showed that on the provincial scale, the urbanisation level and the number of traditional village distribution showed a weak negative correlation with a correlation coefficient of −0.245; to further reveal the correlation between the two in local areas, nine prefectures (cities) in the province were selected for comparison, and we found that the highest degree of correlation was Anshun city, with a significant positive correlation and a coefficient of 0.949, while the remaining regions showed a weak negative, and the remaining regions showed a weak negative correlation. The high number of traditional villages and the high level of urbanisation in Anshun may be related to the fact that most of the inhabitants of villages around Tunbao of Anshun belonged to the Han ethnic group that moved to Pingxi (Tunbao) in the early Ming Dynasty, and this ethnic group persistently inherited the traditional cultural genes of their ancestors during the urbanisation process so that their historical characteristics were preserved and continued.

#### Distribution of the level of economic development and education level

**Figure 10:**
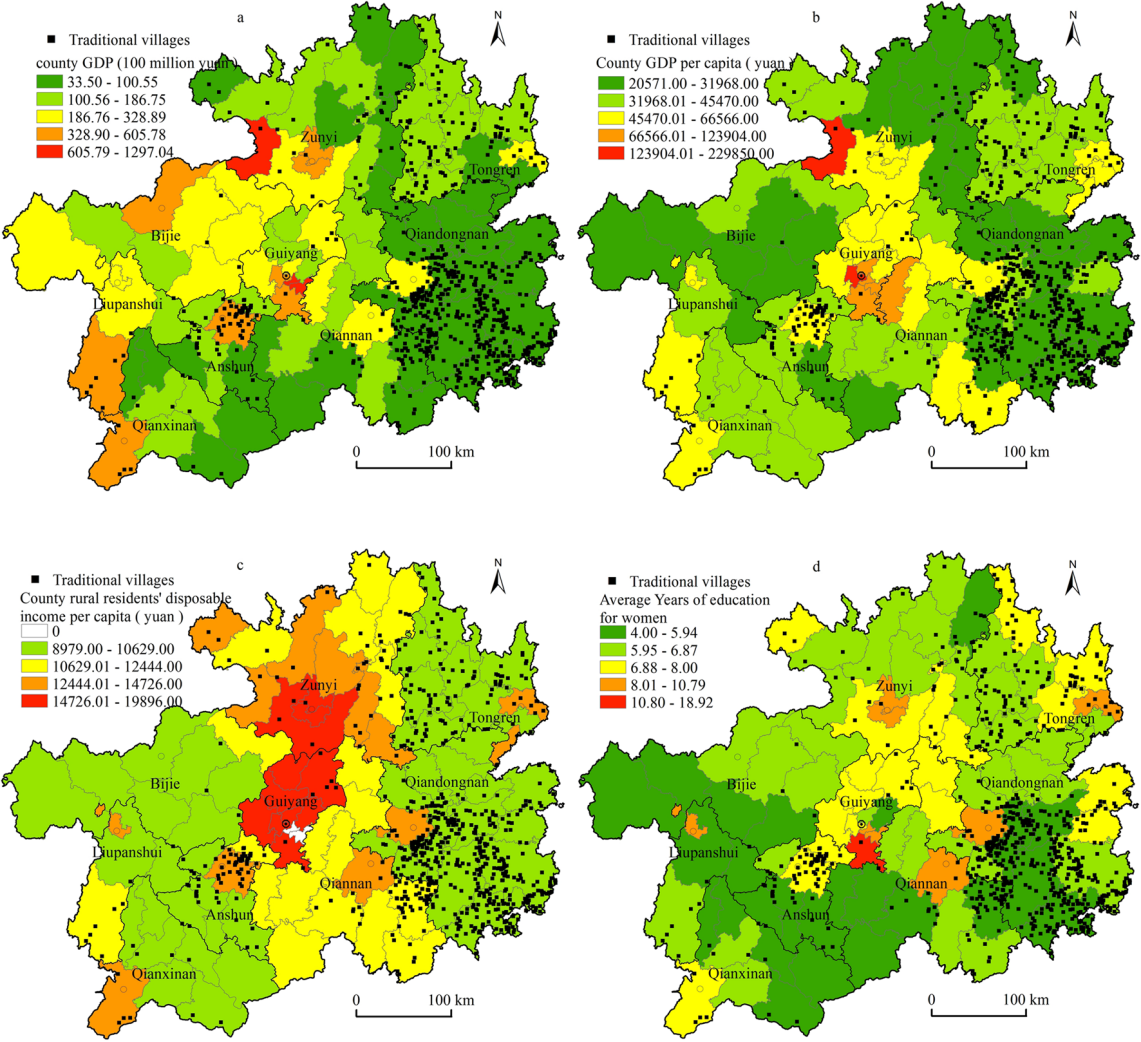
Distribution of traditional villages in terms of economic development and education level (a, b, c, d). Maps were created using ArcGIS10.2 (Environmental Systems Research Institute, USA. https://www.esri.com/).

The spatial distribution maps of county GDP, county GDP per capita, county rural residents' disposable income per capita, and education level were generated by ArcGIS with traditional villages as the unit and spatially overlaid. The results showed that the regions with lower county GDP levels were located in most of Qiandongnan, southern Tongren, southern Qiannan, southern Anshun, northern and southeastern Qianxinan, etc. Traditional villages hadspatial effects that werehighly consistent with the GDP attributes of these regions, especially in Qiandongnan and Tongren (Fig. 10a), and county GDP per capita and traditional villages also showed similar geographic patterns (Fig. 10b). In terms of county, in terms of rural per capita disposable income, Xixiu of Anshun and Bijiang of Tongren hadboth high levels of rural per capita disposable income and a high density of traditional villages, which was related to the fact that these two districts and counties were regional administrative centres and the long-term influence and role of the Tunbao and Tusi cultures, while most of the regions of Qiandongnan and Tongren showed a general pattern of low rural per capita disposable income but a high density of traditional village distribution (Fig. 10c). In terms of education level, by superimposing data related to the number of people with tertiary education and above, the degree of higher education per 100,000 population, and the number of years of education in the county, it was found that the spatial distribution of traditional villages had a significant direct spatial correspondence with the number of years of education, especially the number of years of education for women (Fig. 10d), which may be related to the greater influence of women on family livelihood and children's education, as well as their influence on traditional culture and customs. This may be related to the greater influence of women on household livelihoods and children's education, as well as the more solidified continuity in traditional culture and customs, which indirectly maintain the continuity of traditional villages from an ideological perspective.

### Single driving factor detection of the traditional village distribution

We selected seven independent variables of direct influence factors, such as geomorphology, geology, ecology, land use, spatial location and transportation, ethnic culture, economic conditions and education level, and 35 single factors corresponding to them for driving force detection (Table 4). All the factors passed the significance test. The results showed that the spatial distribution pattern of traditional villages in Guizhou was mainly influenced by the nearest distance to the river and valley, the administrative edge effect index, the geometric edge effect index, the type and number of provincial-level ICHs composed of folklore + traditional music + folk dance + folk art + traditional drama + opera + traditional sports − amusement − acrobatics, etc. The six factors driven by county ethnic language use, had weight q-values that were all greater than 0.30; three factors, such as the full type and number of provincial ICHs in the county, the density of rivers and valleys, and the proportion of paddy fields in the regional area, had a greater influence on the spatial distribution of traditional villages; the vegetation index, the density of paddy field boundary lines, the county forest coverage, the county average traffic density, the proportion of illiterate women in the county, the county female education level, and the thirteen factors, including traffic network line density, county average traffic density, regional forest cover, county GDP, lithology, urbanisation level in 2010, county average elevation, and fault line density, had some influence on the spatial distribution of traditional villages, while the remaining other factors did not significantly influence traditional villages. Overall, the spatial distribution of traditional villages in Guizhou was mostly influenced and controlled by five major factors, such as geomorphology, traditional ethnic culture, locational spatial relationships, agricultural traditional heritage, and education level.

**Table 4:** Driving factors and weights of the spatial distribution of traditional villages.

Primary factor	Secondary factor	*q* value	sort
Geomorphic conditions	Altitude (× 1)	0.021**	30
	County average altitude (× 2)	0.107**	19
	County average surface relief (× 3)	0.061**	25
	County average slope (× 4)	0.081**	23
	County average slope aspect (× 5)	0.085**	21
	The density of rivers and valleys (× 6)	0.225**	**8**
	Nearest distance from river valley (× 7)	0.693**	**1**
Geological conditions	Proportion of carbonate rock and clastic rock in the whole province (× 8)	0.127**	17
	Geological fault line density (× 9)	0.103**	20
Ecological environment	Temperature humidity index (× 10)	0.049**	28
	Vegetation index (× 11)	0.192**	10
	County forest coverage (× 12)	0.187**	11
	Regional forest coverage (× 13)	0.160**	15
Cultivated land use attribute	Proportion of paddy field in the area (× 14)	0.203**	**9**
	Paddy patch fragmentation (× 15)	0.061**	25
	Paddy field boundary line density (× 16)	0.192**	10
	Paddy field surface core density (× 17)	0.107**	19
Space and traffic	Administrative edge effect index (× 18)	0.456**	**3**
	Geometric edge effect index (× 19)	0.394**	**4**
	Traffic network line density (× 20)	0.161**	14
	County average traffic density (× 21)	0.170**	12
	Regional average traffic density (× 22)	0.161**	14
National culture	County-level ethnic diversity index (× 23)	0.058**	26
	County-level national primacy (× 24)	0.063**	24
	County-level ethnic language use (× 25)	0.321**	**6**
	Type and quantity of ICH at county level and provincial level (× 26)	0.262**	**7**
	Type and quantity of ICH at county level and provincial level consisting of folk custom + traditional music + folk dance + folk art + traditional drama + quyi + traditional sports, entertainment, and acrobatics (× 27)	0.331**	**5**
	Type and quantity of ICH at county level and provincial level composed of folk custom + traditional music + folk dance + folk art (× 28)	0.465**	**2**
Economic condition	Urbanization level in 2018 (× 29)	0.083**	22
	Urbanization level in 2010 (× 30)	0.125**	18
	Per capita disposable income in rural areas (× 31)	0.055**	27
	County GDP(× 32)	0.128**	16
	County GDP per capita (× 33)	0.028**	29
Educational level	Percentage of illiterate women in counties (× 34)	0.164**	13
	Average years of education for county women (× 35)	0.164**	13

**Significant difference at the 0.01 level.

### Analysis of combined driving forces of the spatial distribution of traditional villages

To further explore the combinations of constraint factor types that mainly control the distribution of traditional villages and to what extent they are influenced, we used the interactive detection module in the Geodetector to evaluate them. The results showed that the interactive driving factors that strongly influenced the spatial distribution of traditional villages were: nearest distance to river valley (× 7) ∩ type and number of provincial ICHs composed of folklore + traditional music + folk dance + folk art (× 28) (*q* = 0.859), nearest distance to river valley (× 7) ∩ density of river valley (× 6) (*q* = 0.824), nearest distance from river valley (× 7) ∩ vegetation index (× 11) (*q* = 0.805), nearest distance from river valley (× 7) ∩ slope direction (× 5) (q = 0.804), nearest distance from river valley (× 7) ∩ type and number of provincial-level ICHs consisting of folklore + traditional music + folk dance + folk art + traditional drama + opera + traditional sports, amusement and acrobatics (× 27) (*q* = 0.800), nearest distance to river valley ∩ degree of ethnic language use in county (*q* = 0.795), proportion of paddy fields to regional area ∩ nearest distance to river valley (*q* = 0.785), nearest distance to river valley (× 7) ∩ slope (× 4) (*q* = 0.783), nearest distance to river valley (× 7) ∩ full type and number of provincial ICHs in the county (× 26) (*q* = 0.783), the proportion of illiterate females in the county (× 34) ∩ the nearest distance from the river valley (× 7) (*q* = 0.779), the administrative edge effect index (× 18) ∩ the nearest distance from the river valley (× 7) (*q* = 0.776), and the county ethnic diversity index (× 23) ∩ the nearest distance from the river valley (× 7) (*q* = 0.773).

According to the single-factor and interactive detection results, the single-driven and interactive-driven effects on nine items, such as nearest distance to river gorge (× 7), type and number of provincial ICHs in the county (× 26, × 27, × 28), density of river gorge (× 6), edge effect index (× 18, × 19), degree of ethnic language use in the county (× 25), and proportion of paddy fields to the regional area (× 14), showed that their *q* values were generally greater than the other factor weights.

## Discussion

In this paper, the spatial distribution characteristics of traditional villages in mountainous areas and their patterns are inferred more objectively and scientifically through a more microdetection analysis; moreover, the previous qualitative and semiquantitative driving force analysis of traditional villages is expanded to a more concrete quantitative study, and the selection and construction of relevant proxy variable indicators have a positive paradigm effect on the study of traditional villages at different scales, such as watershed, township and village areas. The proposed and measured edge effect index provides a theoretical reference for the scientific expression of the spatial relationship of rural settlement locations; in addition, the geographic detector can better identify seemingly unrelated but intrinsically more significant factors such as river valleys, vegetation structure, rice field landscape, ethnic language and years of education, which will provide a new way of thinking for the discernment and reunderstanding of the problems of traditional village conservation and sustainable development in the mountainous areas of Southwest China.

However, there are many aspects of research on the spatial pattern of traditional villages that need to be deepened. ① Research scale. As the province with the largest distribution of traditional villages in China, most of the current studies focus on the provincial and county areas but not on the scales of watersheds, townships, or villages, which fails to accurately portray the historical evolution and distribution characteristics of villages in a specific space and is not conducive to the systematic and grouped protection of traditional villages. ② Exploration content. Due to the complexity and diversity of human-land systems in mountainous areas, it is necessary to further strengthen the screening, quantification, and detection of such fields or factors as ecosystem structure and function, biodiversity, environmental carrying capacity, ethnic attributes, customs, traditional knowledge, clan religion, village livelihood structure and patterns, and villagers' participation, which have a greater coupling effect with traditional villages. Therefore, we need to construct many natural- and human-related factors to find the right direction for the protection and sustainable use of traditional villages. In addition, The research on the edge effect of traditional villages only discusses "what" and does not delve into "why". Therefore, in the future research process, it is necessary to strengthen the exploration of relevant mechanism research and provide more scientific theoretical basis for the protection of traditional villages. ③ Spatial gene mining. The traditional villages in mountainous areas of Guizhou are mainly formed and evolved based on different ethnic attributes, while the identification of village landscape genes for the cultural lineages of ethnic minorities, military cantons and the Tusi is not covered or not deep enough. The shortcomings of research in spatial genetic diversity differentiation, division and zoning of village geographical types and landscape groups exist, and some excellent traditional cultural genes of harmonious coexistence between humans and nature have not been commensurate with the unique and abundant traditional village resources in the region, and there is much room for research expansion.

## Conclusion

The major conclusions are as follows:

① Traditional villages in Guizhou are, in general, highly aggregated and clustered, with some districts and counties in Qiandongnan, Anshun, and Qiannan as the hotspots of very high primacy and extending to Tongren and Zunyi on the border. More than 90% of the traditional villages have significant edge effects and have the distinctive feature of crossing the boundaries of 2–4 county-level administrative units.
② A total of 98.07% of traditional villages are distributed in the range of 250–1500 m above sea level, and the overall spatial density decreases with increasing altitude, is "dense in the east and sparse in the west", and has the characteristic of being far from the central city of the provincial capital; the average surface undulation of traditional villages is higher than that of natural villages, and except for the Anshun area, the spatial density of areas with high surface undulation is higher. The number of traditional villages and their gathering degree are higher in clastic rock areas than in carbonate rock areas, and more than half of them are distributed in the 1500 m buffer zone of the fault line, which is mostly extended in the northeast‒southwest direction; the waterfront characteristics of the villages are remarkable, and they are cool and comfortable. The spatial density, clustering, surface undulation, sunwardness, and waterfront of traditional villages are significantly different from those of nonkarst substrates.
③ Miao, Dong, Buyi, Shui, Tujia, Gelao, and other minority cultures and historical cultural villages represented by Tunbao (military canton) and Tusi culture constitute the main body of traditional village distribution in Guizhou; they have significant direct spatial correspondence with the degree of minority language use, the type and number of ICH, the distribution of rice fields, and the number of years of female education and form a low-low, low–high, low–high, and high-high relationship with transportation and urbanisation.
④ The spatial distribution pattern of traditional villages is mostly controlled by nine single drivers, such as nearest distance to the river valley (× 7), provincial ICH types and numbers in the county (× 26, × 27, × 28), river valley density (× 6), edge effect index (× 18, × 19), degree of ethnic language use in the county (× 25), and proportion of paddy fields to the regional area (× 14), and their combined effects. Among them, the closest distance to the river and valley is dominant in the spatial distribution of traditional villages, further interpreting that easy access to water and water use is the preferred choice of location for human settlements. The type and number of ICH, and the degree of ethnic language use map the cultural identity of traditional villages, which is the centripetal force for their sustainable preservation. Furthermore, the edge effect indicates that traditional villages are located in specific spaces far from or less exposed to the impact of modern civilisation. As a special and highly stable artificial wetland ecosystem, rice paddies, with their "double harvest in one season", "multiple harvest in one season" or "harvest in all seasons" farming attributes, greatly enhance the land yield and continuously nourish the community. It is a special and highly stable artificial wetland ecosystem that greatly enhances land productivity and continues to support villagers for generations.

### Supplementary Information

Supplementary Information 1.

Supplementary Information 2.

## Data Availability

The datasets used and/or analysed during the current study are available from the corresponding author upon reasonable request.

## Abbreviations

GIS: Geographic information system
ICH: Intangible cultural heritage
GDP: Gross domestic product
